# Probability maps classify ischemic stroke regions more accurately than CT perfusion summary maps

**DOI:** 10.1007/s00330-022-08700-y

**Published:** 2022-03-31

**Authors:** Daan Peerlings, Fasco van Ommen, Edwin Bennink, Jan W. Dankbaar, Birgitta K. Velthuis, Bart J. Emmer, Jan W. Hoving, Charles B. L. M. Majoie, Henk A. Marquering, Hugo W. A. M. de Jong

**Affiliations:** 1 Department of Radiology, University Medical Center Utrecht, Utrecht, 3584CX The Netherlands; 2 Image Sciences Institute, University Medical Center Utrecht, Utrecht, 3584CX The Netherlands; 3 Department of Radiology and Nuclear Medicine, Amsterdam University Medical Centers, location Academic Medical Center, Amsterdam, 1105AZ The Netherlands

**Keywords:** Brain ischemia, Logistic models, Perfusion imaging, Stroke, Tomography, X-ray computed

## Abstract

**Objectives:**

To compare single parameter thresholding with multivariable probabilistic classification of ischemic stroke regions in the analysis of computed tomography perfusion (CTP) parameter maps.

**Methods:**

Patients were included from two multicenter trials and were divided into two groups based on their modified arterial occlusive lesion grade. CTP parameter maps were generated with three methods—a commercial method (ISP), block-circulant singular value decomposition (bSVD), and non-linear regression (NLR). Follow-up non-contrast CT defined the follow-up infarct region. Conventional thresholds for individual parameter maps were established with a receiver operating characteristic curve analysis. Probabilistic classification was carried out with a logistic regression model combining the available CTP parameters into a single probability.

**Results:**

A total of 225 CTP data sets were included, divided into a group of 166 patients with successful recanalization and 59 with persistent occlusion. The precision and recall of the CTP parameters were lower individually than when combined into a probability. The median difference [interquartile range] in mL between the estimated and follow-up infarct volume was 29/23/23 [52/50/52] (ISP/bSVD/NLR) for conventional thresholding and was 4/6/11 [31/25/30] (ISP/bSVD/NLR) for the probabilistic classification.

**Conclusions:**

Multivariable probability maps outperform thresholded CTP parameter maps in estimating the infarct lesion as observed on follow-up non-contrast CT. A multivariable probabilistic approach may harmonize the classification of ischemic stroke regions.

**Key Points:**

• *Combining CTP parameters with a logistic regression model increases the precision and recall in estimating ischemic stroke regions*.

• *Volumes following from a probabilistic analysis predict follow-up infarct volumes better than volumes following from a threshold-based analysis*.

• *A multivariable probabilistic approach may harmonize the classification of ischemic stroke regions*.

**Supplementary Information:**

The online version contains supplementary material available at 10.1007/s00330-022-08700-y.

## Introduction

Since endovascular treatment revolutionized acute ischemic stroke care, baseline imaging has become ever more relevant to select patients for treatment [1–3]. Personalized selection criteria may be provided by computed tomography perfusion (CTP) imaging.

For a patient with acute ischemic stroke, a CTP scan should estimate the irreversibly damaged tissue (i.e., the infarct core) and the salvageable tissue (i.e., the penumbra), which together form the total hypoperfused region. To classify these regions, the CTP scan is processed with dedicated software to produce four perfusion maps: the cerebral blood flow (CBF), cerebral blood volume (CBV), mean transit time (MTT), and time to peak (TTP/Tmax). Subsequently, a predefined threshold can be applied to outline the ischemic core and the penumbra.

However, different approaches in CTP processing software and analysis between vendors have led to a variety of threshold values for ischemia in stroke imaging (Table 1). These different thresholds may partly contribute to the discordance between vendors in CTP results, hampering multicenter CTP studies. A standardized classification method could increase harmony in CTP results between different processing methods.

**Table 1:** Clinical definitions of ischemic core and penumbra that are currently implemented in varying commercially available perfusion software packages. CBF cerebral blood flow, CBV cerebral blood volume, MTT mean transit time, and TTP/Tmax time to peak. Values relative to the opposite hemisphere are indicated by an “r”

Software	Ischemic core	Penumbra
IntelliSpace Portal (Philips Healthcare)	CBV < 2.0 mL/100g & rMTT > 150%	rMTT > 150%
Syngo.via (Siemens Healthineers)	CBV < 1.2 mL/100g	CBF < 27.0 mL/100g/min
Vitrea (Toshiba/Canon Medical Systems)	rCBV < 41%	TTP > 6.8 s
RAPID (iSchemaView)	rCBF < 30%	Tmax > 6 s

Probabilistic classification of ischemic stroke regions has been proposed as an alternative to threshold-based classification [4, 5]. Probability maps can combine parameters and can indicate certainty of ischemia [4, 6]. Volumes obtained from probability maps were already validated against conventional threshold-based volumes for probability models that include a single perfusion parameter [4]. However, the chosen perfusion parameter may still differ between vendors and the disregarded maps may contain additional—unused—information. Also, a multivariable probabilistic classification has been compared to single parameter thresholding, but only for different CTP software between the two classification methods [7]. Probabilistic classification has not yet been compared to threshold-based classification for a probability map that combines multiple perfusion parameters readily available within any single CTP software.

This study tests the hypothesis that a multivariable probabilistic analysis of perfusion maps is superior to single variable thresholding in predicting the ischemic core and total hypoperfused region.

## Methods

### Acquisition of imaging data

Both the DUtch acute STroke (DUST) study, in which fourteen stroke centers participated, and the Multicenter Randomized Clinical Trial of Endovascular Treatment for Acute Ischemic Stroke in the Netherlands (MRCLEAN), in which seventeen stroke centers participated, contributed their data to this study [8, 9]. All included DUST participants (*n* = 182) and included MRCLEAN participants (*n* = 43) gave informed consent for the use of their clinical and imaging data.

The DUST study protocol design describes acquisition of the admission CTP scan at 80 kVp and 150 mAs on 40- to 320-detector CT scanners (GE Healthcare, Philips, Siemens, Toshiba) with a 2-s interval for a duration of 50 s and reconstructed to a slice thickness of 5 mm. The advised injection protocol was a 40 mL contrast bolus injected at a rate of 6 mL/s followed by a saline flush of 40 mL injected at a rate of 6 mL/s. Patients eligible for treatment received intravenous thrombolysis, intra-arterial thrombolysis, and/or mechanical thrombectomy. For this study, the necessary follow-up imaging consisted of a non-contrast CT (NCCT) as well as a CT angiography (CTA) scan within 3 days.

In the MRCLEAN trial, centers could adhere to their own acquisition and injection protocol. Patients eligible for treatment received intravenous thrombolysis, intra-arterial thrombolysis, and/or mechanical thrombectomy. The necessary follow-up imaging again consisted of a NCCT as well as a CTA scan and was acquired after 24 h.

### Processing of imaging data

For the data from the DUST study, a radiologist with 6 years of experience manually segmented the follow-up NCCT of each patient to define the follow-up infarct region. In the MRCLEAN trial, the follow-up NCCT of each patient was segmented automatically using a convolutional neural network [10]. Because our study compared two classification methods, any inconsistencies in the follow-up infarct regions were the same for both these methods.

The total patient population (*n* = 225) was divided into a patient group with successful recanalization (REC, *n* = 166) and a patient group with persistent occlusion (OCC, *n* = 59), based on the modified arterial occlusive lesion (mAOL) grade determined from the follow-up CTA (REC: mAOL grade 3; OCC: mAOL grade 0 and 1) [11]. Subsequently, each patient group was divided 2:1 into a REC/OCC training patient group (*n* = 110/*n* = 39) and a REC/OCC test patient group (*n* = 56/*n* = 20).

Patients were divided into a REC and OCC patient group because the segmentation on the follow-up NCCT should resemble the infarct core at the time of admission imaging for the REC patient group (since the recanalization should have saved the penumbra) whereas the segmentation on the follow-up NCCT should resemble the total hypoperfused region at the time of admission imaging for the OCC patient group (since the occlusion should have infarcted the penumbra). Hence, the REC patient group was used to train and test the classification of the infarct core, whereas the OCC patient group was used to train and test the classification of the total hypoperfused region.

To assess the robustness and universality of our method, perfusion maps were generated with three perfusion processing methods, all providing a CBF, CBV, MTT, and TTP map. The first is a commercial method in which the CTP scan was analyzed with the arrival-time-sensitive algorithm in IntelliSpace Portal (ISP; Brain Perfusion, IntelliSpace Portal 10.1, Philips Healthcare). The second is an in-house developed method, which uses a block-circulant singular value decomposition (bSVD) [12] algorithm. The third is an in-house model-based non-linear regression (NLR) method [13].

Prior to perfusion analysis, the CTP scans were processed the same way for both in-house methods (bSVD and NLR), as described previously [14]. For ISP, the IntelliSpace Portal Brain Perfusion application was used to filter the CTP image data as well as to automatically select the arterial input function (AIF) and venous output function (VOF). All further data processing and analysis were carried out with MATLAB (MATLAB, R2019b: The Mathworks Inc.).

### Determining thresholds

To determine thresholds, we followed (and refer to) the procedure on which the current clinical thresholds of ISP are based [15]. To summarize, a receiver operating characteristic (ROC) curve is produced for each perfusion parameter. The perfusion parameter yielding the largest AUC of its ROC curve is chosen as the parameter to define either the ischemic core (for the REC training patient group) or the total hypoperfused region (for the OCC training patient group). The threshold value for this perfusion parameter is then found by maximizing the Youden index [16].

### Determining probability models

To determine the probability models, we performed logistic regression by maximum likelihood estimation on all four perfusion parameters with follow-up tissue outcome as a response variable. This resulted in a logistic model for the ischemic core (from the REC training patient group) and for the total hypoperfused region (from the OCC training patient group):

$$ P\left(\mathrm{CORE}\right)=1/\left(1+{e}^{\left({C}_{\mathrm{INT},\kern0.5em \mathrm{REC}}+{C}_{\mathrm{CBF},\mathrm{REC}}\times \mathrm{CBF}+{C}_{\mathrm{CBV},\mathrm{REC}}\times \mathrm{CBV}+{C}_{\mathrm{MTT},\mathrm{REC}}\times \mathrm{MTT}+{C}_{\mathrm{TTP},\mathrm{REC}}\times \mathrm{TTP}\right)}\right), $$

$$ P\left(\mathrm{HYPOPERFUSED}\right)=1/\left(1+{e}^{\left({C}_{\mathrm{INT},\mathrm{OCC}}+{C}_{\mathrm{CBF},\mathrm{OCC}}\times \mathrm{CBF}+{C}_{\mathrm{CBV},\mathrm{OCC}}\times \mathrm{CBV}+{C}_{\mathrm{MTT},\mathrm{OCC}}\times \mathrm{MTT}+{C}_{\mathrm{TTP},\mathrm{OCC}}\times \mathrm{TTP}\right)}\right). $$

Once the coefficients for the intercept (*C*_INT_), the CBF (*C*_CBF_), the CBV (*C*_CBV_), the MTT (*C*_MTT_), and the TTP (*C*_TTP_) were determined from the training patient groups, the CBF, CBV, MTT, and TTP of a voxel gave the probability P(CORE) that this voxel belonged to the ischemic core (based on the REC training patient group) and the probability P(HYPOPERFUSED) that this voxel belonged to the total hypoperfused region (based on the OCC training patient group). Calculating these probabilities for all voxels resulted in a probability map for the ischemic core and a probability map for the total hypoperfused region.

### Training data set

The training data set was prepared the same way for both the ROC curve analysis and the logistic regression analysis. Because the ROC curve can show bias towards the majority class in imbalanced data (in our case the class of healthy voxels against the class of ischemic voxels) [17], the training data set was limited to the collection of parenchymal voxels in the ischemic hemisphere of slices with a segmentation of the follow-up infarct region. To minimize the impact of high leverage voxels on logistic regression (in our case voxels with normal perfusion in the segmented region on the ground truth map and voxels with reduced perfusion outside this region) [18], voxels with an outlier in one of the perfusion parameters were removed. An outlier was defined as a data point more than 1.5 times the interquartile range below the first quartile or above the third quartile [19].

### Determining volumes

A threshold-based volume followed from a summary map by summing the voxel volumes of all voxels in a classified region. Before determining a volume, the summary map was morphologically opened and then morphologically closed, both with a spherical structure element of 5 mm in diameter, to reduce noise artefacts.

A probabilistic volume follows from a probability map by summing the probabilities, multiplied by the voxel volume, of the left and right hemisphere separately and taking the absolute difference between these two sums. Noise artefacts are automatically accounted for in the comparison between the two hemispheres.

### Classification performance

The classification performance of both methods was assessed on the level of voxels as well as on the level of patients. On the level of voxels, a precision-recall curve was produced for each perfusion parameter and for the probability. These curves show the precision and the recall for different thresholds of a perfusion parameter or of the probability. On the level of patients, the predicted threshold-based volume and the predicted probabilistic volume were compared to the follow-up infarct volume for each patient. Both assessments were carried out on the total ischemic hemisphere.

A precision-recall curve was used to visualize classification performance because of considerable class imbalance between the ischemic and healthy tissue in the total ischemic hemisphere [17]. In the context of classifying ischemic regions, the precision is the percentage of the classified region that is truly ischemic core or hypoperfused and the recall is the percentage of the true ischemic core or hypoperfused region that is correctly classified.

The predicted volume was compared to the follow-up infarct volume because the final infarct volume is a principal predictor of functional outcome [20–22]. The volume difference between the predicted volume and the ground truth volume was defined as the predicted volume minus the ground truth volume. A boxplot of the volume difference was made for each patient group (i.e., REC and OCC), each CTP processing method (i.e., ISP, bSVD, and NLR), and each classification method (i.e., threshold-based and probabilistic). The mean volume differences of the threshold-based classification and probabilistic classification were compared with a paired *t*-test for each patient group and for each CTP processing method. The level of significance was defined as a two-tailed *p* < 0.05.

## Results

### Threshold-based classification of ischemic regions

**Fig. 1:**
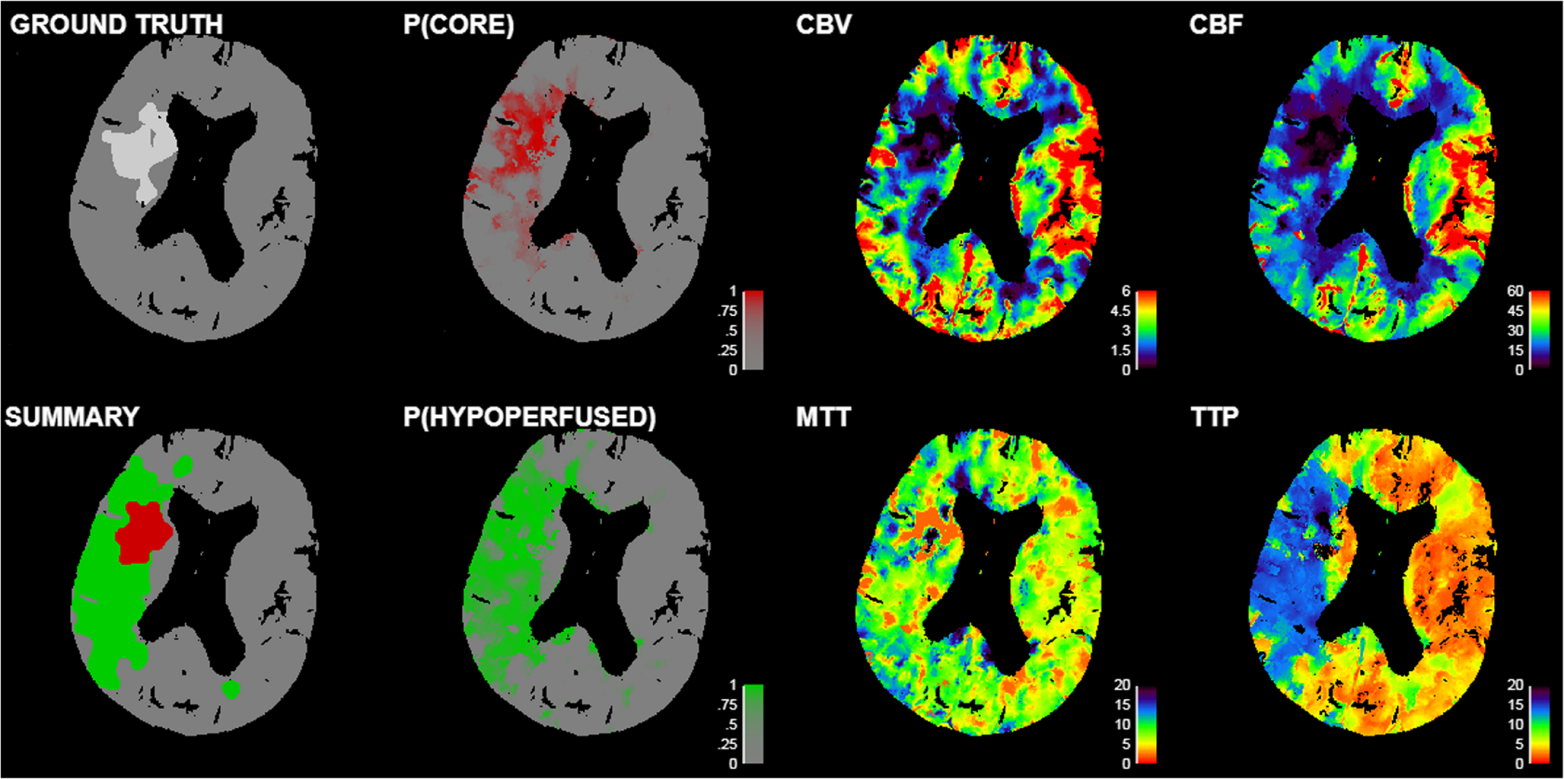
The ground truth map (i.e., manual segmentation from a follow-up non-contrast CT scan, in this case from the REC patient group), summary map (obtained from thresholding according to Table 2, in this case for the bSVD processing method), the probability map P(CORE) in case of successful recanalization (REC; obtained from the logistic model in Table 3, in this case for the bSVD processing method), the probability map P(HYPOPERFUSED) in case of persistent occlusion (OCC; obtained from the logistic model in Table 3, in this case for the bSVD processing method), and the perfusion maps. The cerebral blood flow (CBF) is in mL/100g/min, the cerebral blood volume (CBV) is in mL/100g, the mean transit time (MTT) is in seconds, and the time to peak (TTP) is in seconds

Based on the REC training patient group (to acquire the optimal threshold for the ischemic core), the CBF was the parameter with the highest AUC of its ROC curve for each processing method (Table 2). Based on the OCC training patient group (to acquire the optimal threshold for the total hypoperfused region), the MTT had the highest AUC of its ROC curve for the ISP processing method and the TTP had the highest AUC of its ROC curve for both in-house processing methods (Table 2). For these parameters, the threshold value was determined by maximizing the Youden index. Figure 1 shows an example summary map.

**Table 2:** Thresholds following from a receiver operating characteristic (ROC) curve analysis for three processing methods (ISP, bSVD, NLR) based on a training patient group with successful recanalization (REC) and a training patient group with persistent occlusion (OCC). CBF cerebral blood flow, MTT mean transit time, TTP time to peak, and AUC area under the curve

Method	Training patient group	Threshold	AUC of ROC curve	Youden’s index
ISP	REC	CBF < 14.0 mL/100g/min	0.68	0.29
	OCC	MTT > 11.0 s	0.75	0.44
bSVD	REC	CBF < 9.0 mL/100g/min	0.74	0.38
	OCC	TTP > 6.0 s	0.79	0.49
NLR	REC	CBF < 10.0 mL/100g/min	0.78	0.41
	OCC	TTP > 6.5 s	0.82	0.54

### Probabilistic classification of ischemic regions

Table 3 shows the coefficients from a logistic regression analysis to acquire probability maps. The positive model coefficients for the CBF and CBV reflect that the CBF and CBV decrease in an ischemic region. The negative model coefficients for the MTT and TTP reflect that the MTT and TTP increase in an ischemic region. For the CBF and CBV, the coefficient from the REC patient group is higher than the coefficient from the OCC patient group for each of the three processing methods. For the MTT and TTP, the coefficient from the OCC patient group is lower than the coefficient from the REC patient group for each of the three processing methods. This implies that the CBF and CBV were more important for predicting the ischemic core, whereas the MTT and TTP were more important for predicting the total hypoperfused region. Figure 1 shows an example probability map in case of successful recanalization, and in case of persistent occlusion.

**Table 3:** Coefficients following from a logistic regression analysis for three processing methods (ISP, bSVD, NLR) based on a training patient group with successful recanalization (REC) and a training patient group with persistent occlusion (OCC). The coefficient for the CBF (C_CBF_) is in (mL/100g/min)^−1^, the coefficient for the CBV (C_CBV_) is in (mL/100g)^−1^, the coefficient for the MTT (C_MTT_) is in (seconds)^−1^, and the coefficient for the TTP (C_TTP_) is in (seconds)^−1^

Method	Patient group	*C*_INT_	*C*_CBF_	*C*_CBV_	*C*_MTT_	*C*_TTP_
ISP	REC	2.31	0.06	0.26	−0.09	−0.08
	OCC	3.98	0.04	0.12	−0.21	−0.17
bSVD	REC	2.58	0.13	0.57	−0.14	−0.32
	OCC	3.09	0.08	0.30	−0.16	−0.38
NLR	REC	3.45	0.14	0.66	−0.15	−0.39
	OCC	4.16	0.09	0.28	−0.17	−0.48

### Classification performance

**Fig. 2:**
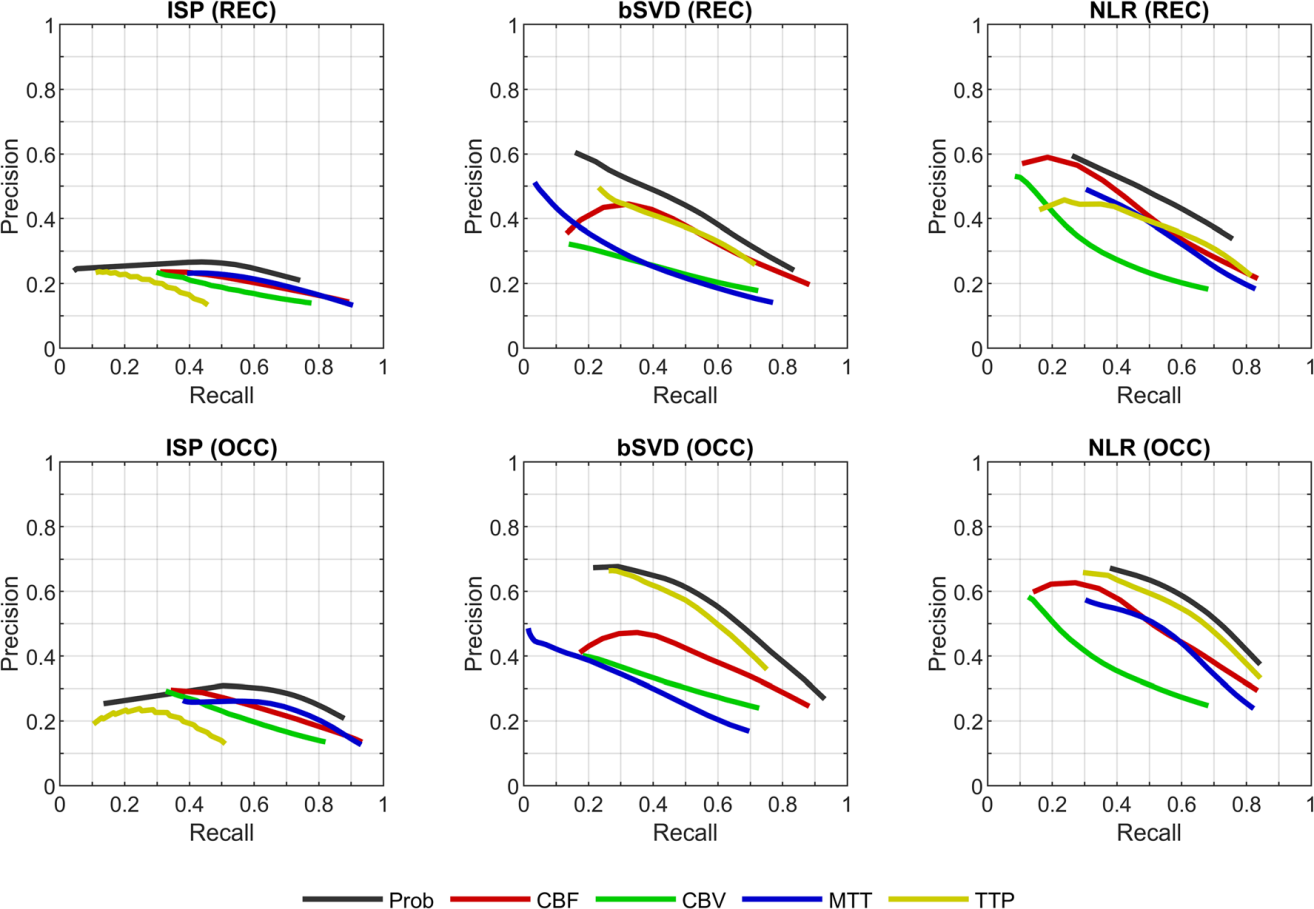
Precision-recall curves of the perfusion and probability maps for three processing methods (ISP, bSVD, NLR) following from a test patient group with successful recanalization (REC) and a test patient group with persistent occlusion (OCC). CBF stands for cerebral blood flow, CBV for cerebral blood volume, MTT for mean transit time, TTP for time to peak, and Prob for the probability (corresponding to the patient group)

The precision-recall curve of the probability generally lies above the precision-recall curves of the perfusion parameters (Fig. 2), indicating a better classification performance. For ISP, low values of the CBF and CBV may have a higher precision in predicting the total hypoperfused region (i.e., in the OCC patient group) than the probability at the same (low) level of recall. Clinically, however, these low values of the CBF and CBV are not so relevant for predicting the total hypoperfused region because of the low recall.

For ISP, the curve for the CBF was calculated for 1 to 40 mL/100g/min in steps of 2 mL/100g/min, the curve for the CBV for 0.1 to 4.0 mL/100g in steps of 0.2 mL/100g, the curve for the MTT for 25 to 5 s in steps of 1 s, the curve for the TTP for 15 to 5 s in steps of 1 s, and the curve for the probability for 95 to 5% in steps of 5%. For the in-house processing methods, the curve for the CBF was calculated for 1 to 20 mL/100g/min in steps of 1 mL/100g/min, the curve for the CBV for 0.1 to 2.0 mL/100g in steps of 0.1 mL/100g, the curve for the MTT for 25 to 5 s in steps of 1 s, the curve for the TTP for 15 to 5 s in steps of 0.5 s, and the curve for the probability for 95 to 5% in steps of 5%.

**Fig. 3:**
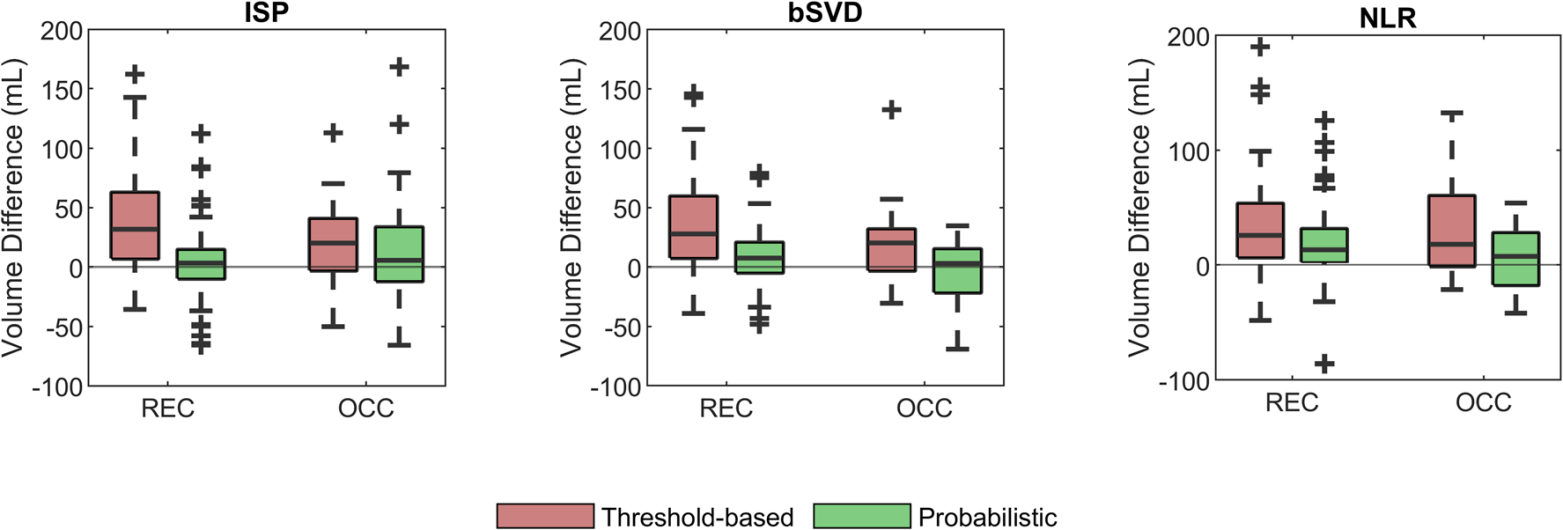
Boxplots of the volume difference between the ground truth volume and the predicted volume (either threshold-based or probabilistic) for three processing methods (ISP, bSVD, NLR) following from a test patient group with successful recanalization (REC) and a test patient group with persistent occlusion (OCC)

Threshold-based classification led to an overall overestimation of the follow-up infarct volume within the test patient groups. For the REC and OCC test patient groups combined, the median volume difference [Q1, Q3] in mL was 29 [4, 56]/23 [3, 53]/23 [4, 56] (ISP/bSVD/NLR) for threshold-based classification and was 4 [−10, 21]/6 [−7, 18]/11 [0, 30] (ISP/bSVD/NLR) for probabilistic classification. For each test patient group separately, the volume difference following the probabilistic classification was lower than from the threshold-based classification (Fig. 3).

Between threshold-based classification and probabilistic classification, the mean ischemic core volume difference differed significantly for each processing method (*p* < 0.001). The mean hypoperfused region volume difference differed significantly for bSVD (*p* = 0.003) as well as for NLR (*p* = 0.002) but not for ISP (*p* = 0.24). A scatter plot of the volumes and a Bland-Altman plot of the volumes can be found in the Supplementary Material.

## Discussion

Our results show that combining perfusion parameters in a logistic model improved the precision-recall curve and that probabilistic volumes were significantly more accurate than threshold-based volumes in estimating the infarct volume on follow-up non-contrast CT obtained within 3 days. This study suggests that multivariable probability maps classify ischemic stroke regions more accurately than CTP summary maps.

Fixed single parameter thresholds do not use the available information to its full potential, because of their limitation to incorporate multiple (perfusion) parameters as well as their limitation to show the certainty of predicted ischemia; a voxel is classified as either completely healthy or not, regardless of its proximity to the defined threshold or the value of the other perfusion parameters [4, 6, 23]. Moreover, the existence of a universal pathophysiological cutoff value to determine the final tissue state is questionable due to oversimplification [4, 5].

For a logistic model with four perfusion parameters, we showed that the precision and recall of the probability map are better than that of the individual perfusion maps. The precision-recall curve of a probability map that follows from a logistic model with a single perfusion parameter is identical to the precision-recall curve of the perfusion parameter itself. Therefore, the inclusion of multiple perfusion parameters in a logistic model improved the model. However, we have not compared the probabilistic volumes following from our multivariable logistic model with probabilistic volumes following from single variable logistic models. Instead, we have tested the multivariable logistic model against single variable thresholding because in the current clinical setting, thresholds are typically applied to single perfusion maps.

A strength of our study is that data was used from two different multicenter trials with multiple CT vendors and was divided into a training and test patient group, which gives generalizable results. The data also included small and large follow-up infarct volumes. Our analysis has strengths as well. First, the validation with three CTP processing methods demonstrated the translatability of our method of probabilistic classification. Second, we compared the predicted volumes of both methods next to a voxel-wise comparison—as represented by the precision-recall curve—because the final infarct volume is characterized as pivotal in determining functional outcome [20, 21, 24].

Several limitations to our study should be noted. First, NCCT was used as follow-up imaging method since better methods such as diffusion-weighted MR imaging was not generally available for our data. For patients from the MRCLEAN trial, the infarct was sometimes poorly visible on the follow-up NCCT after 24 h. Additionally, the centers in the MRCLEAN trial could adhere to their own acquisition and injection protocol, which introduces variability to the CTP results [25–27]. For the REC patient group, the ischemic core may have grown between the time of admission imaging and recanalization, especially for patients who received intravenous thrombolytic therapy. As a result, the ground truth maps for the REC patient group may cover substantial parts of the penumbra at the time of imaging. The resulting probability maps should therefore be interpreted as an estimation of the ischemic core at the time of reperfusion [6]. Also, for all patients, the ground truth map could be influenced by brain shift due to edema.

There are weaknesses to our analysis as well. First, class imbalance, although minimized by the choice of our sample space, can lead to low predictive accuracy for the class of ischemic voxels in both classification methods [17]. Second, regarding probabilistic classification in specific, the decision to include all four perfusion maps may not be optimal for logistic regression because of the correlation between the perfusion parameters. Third, we interpreted the probabilities as volume fractions and estimated the ischemic core and total hypoperfused region volumes by taking the difference between both hemispheres, but this approach may leave room for improvement. Fourth, relative values of the perfusion parameters were not studied both because relative perfusion parameter maps could not be exported from ISP and because no clear definition of relative values exists.

## Conclusion

Multivariable probability maps outperform conventional CTP summary maps in estimating the follow-up infarct lesion, as observed on follow-up non-contrast CT obtained within 3 days. Clinically, an improved classification benefits the selection to treat acute ischemic stroke patients. Probability maps may provide an improved and standardized classification of ischemic regions in CTP stroke imaging.

## **Supplementary information**

ESM (DOCX 432 kb)

## Abbreviations

AIF: Arterial input function
bSVD: Block-circulant singular value decomposition
CBF: Cerebral blood flow
CBV: Cerebral blood volume
CTA: CT angiography
CTP: Computed tomography perfusion
DUST: DUtch acute STroke (study)
ISP: IntelliSpace Portal
mAOL: Modified arterial occlusive lesion (grade)
MRCLEAN: Multicenter Randomized Clinical Trial of Endovascular Treatment for Acute Ischemic Stroke in the Netherlands
MTT: Mean transit time
NCCT: Non-contrast CT
ROC: Receiver operating characteristic (curve)
TTP: Time to peak
VOF: Venous output function

## Declarations

### Guarantor

The scientific guarantor of this publication is H. W. A. M. de Jong.

### Statistics and Biometry

No complex statistical methods were necessary for this paper.

### Study subjects or cohorts overlap

Some study subjects or cohorts have been previously reported in

van Seeters, T., Biessels, G. J., van der Schaaf, I. C., Dankbaar, J. W., Horsch, A. D., Luitse, M. J., ... & Velthuis, B. K. (2014). Prediction of outcome in patients with suspected acute ischaemic stroke with CT perfusion and CT angiography: the Dutch acute stroke trial (DUST) study protocol. *BMC neurology*, *14*(1), 1-8.

Van Der Hoeven, E. J., Dankbaar, J. W., Algra, A., Vos, J. A., Niesten, J. M., Van Seeters, T., ... & Velthuis, B. K. (2015). Additional diagnostic value of computed tomography perfusion for detection of acute ischemic stroke in the posterior circulation. *Stroke*, *46*(4), 1113-1115.

Hamming, A. M., van Walderveen, M. A., Mulder, I. A., van der Schaaf, I. C., Kappelle, L. J., Velthuis, B. K., ... & Algra, A. (2019). Circle of Willis variations in migraine patients with ischemic stroke. *Brain and behavior*, *9*(3), e01223.

van Seeters, T., Biessels, G. J., Kappelle, L. J., Van Der Schaaf, I. C., Dankbaar, J. W., Horsch, A. D., ... & Schonewille, W. J. (2015). The prognostic value of CT angiography and CT perfusion in acute ischemic stroke. *Cerebrovascular diseases*, *40*(5-6), 258-269.

van Seeters, T., Biessels, G. J., Kappelle, L. J., van der Schaaf, I. C., Dankbaar, J. W., Horsch, A. D., ... & Schonewille, W. J. (2016). CT angiography and CT perfusion improve prediction of infarct volume in patients with anterior circulation stroke. *Neuroradiology*, *58*(4), 327-337.

Horsch, A. D., Bennink, E., van Seeters, T., Kappelle, L. J., van der Graaf, Y., Mali, W. P., ... & DUST Investigators. (2018). Computed tomography perfusion derived blood-brain barrier permeability does not yet improve prediction of hemorrhagic transformation. *Cerebrovascular Diseases*, *45*(1-2), 26-32.

Berkhemer O. A., Fransen P. S. S., Beumer D., … & MRCLEAN Investigators (2015) A randomized trial of intraarterial treatment for acute ischemic stroke. *N Engl J Med 372:11–20.* 10.1056/nejmoa1411587

Fransen P. S. S., Beumer D., Berkhemer O. A., … & MRCLEAN Investigators (2014). MR CLEAN, a multicenter randomized clinical trial of endovascular treatment for acute ischemic stroke in the Netherlands: Study protocol for a randomized controlled trial. *Trials 15:.* 10.1186/1745-6215-15-343

### Methodology

• retrospective

• observational

• multicenter study
