# Supplementary material for: Probability maps classify ischemic stroke regions more accurately than CT perfusion summary maps
Source: Eur Radiol. 2022 Mar 31;32(9):6367–75. doi: 10.1007/s00330-022-08700-y (PMC9381605; doi:10.1007/s00330-022-08700-y)
Supplement: Supplementary file 1 — ESM (DOCX 432 kb) [file 330_2022_8700_MOESM1_ESM.docx]

Supplementary material


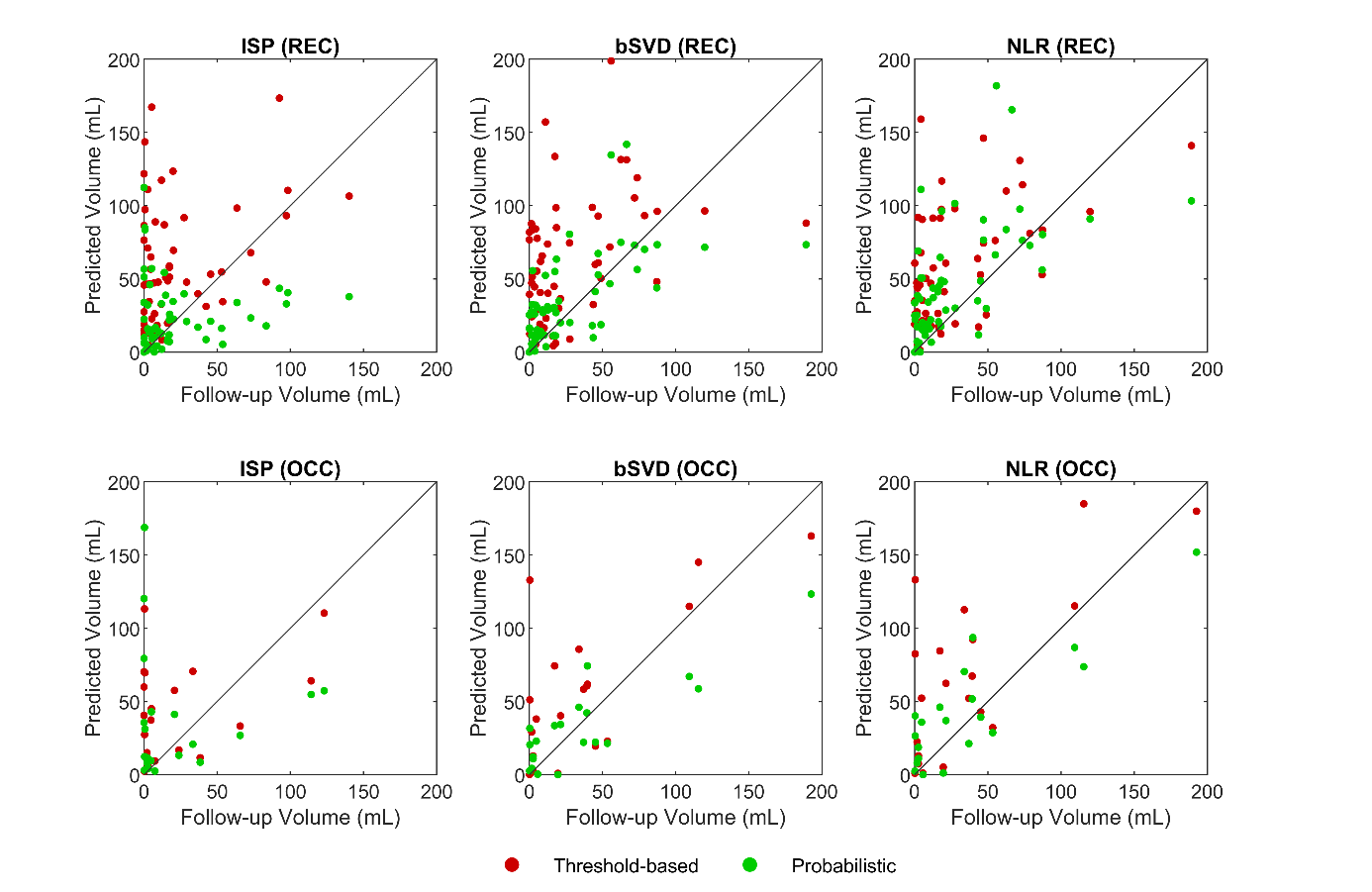


Figure 4

Scatter plot of threshold-based and probabilistic volumes for three processing methods (ISP, bSVD, NLR) following from a test patient group with successful recanalization (REC) and a test patient group with persistent occlusion (OCC).


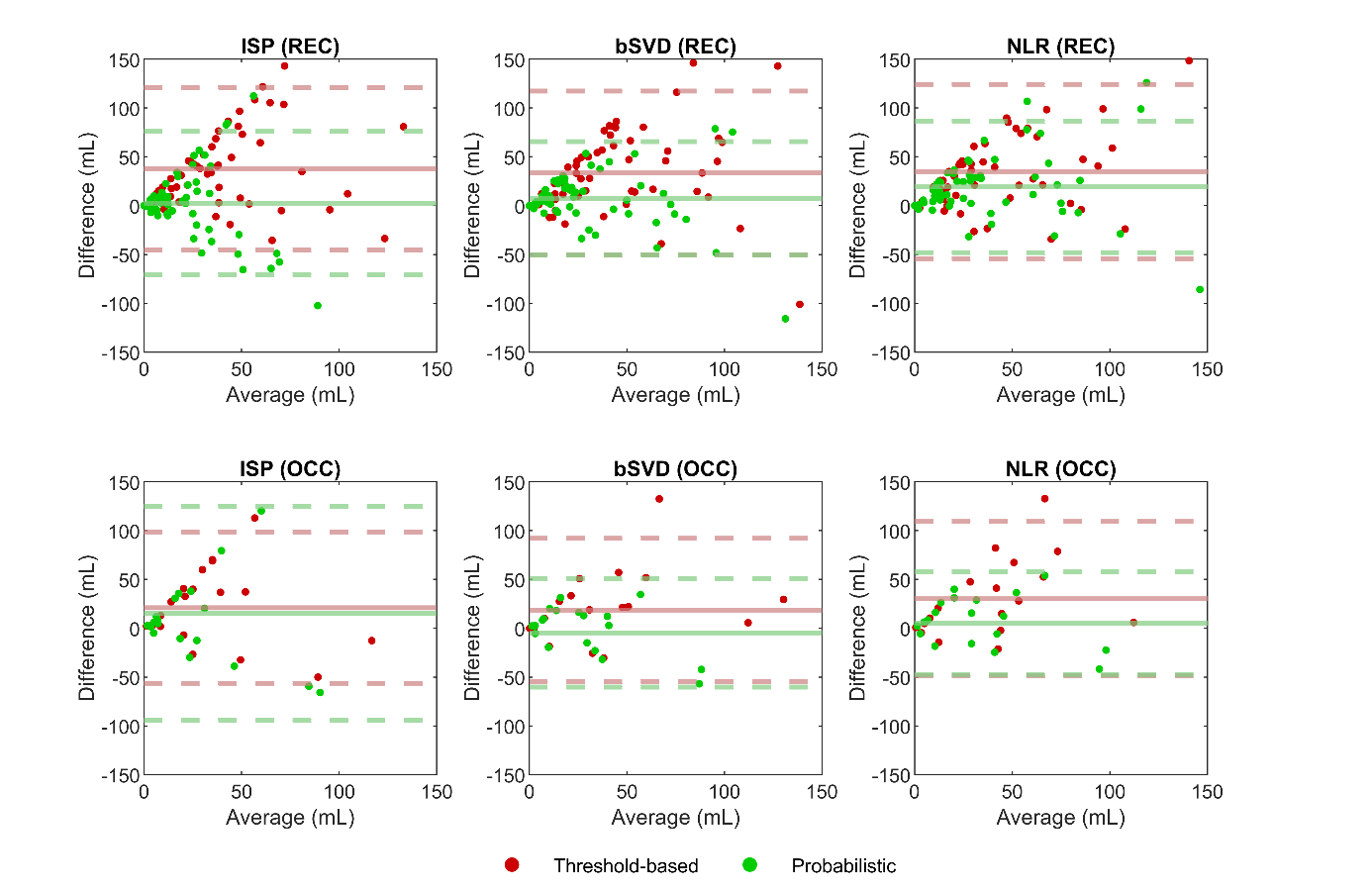


Figure 5

Bland-Altman plot of threshold-based and probabilistic volumes for three processing methods (ISP, bSVD, NLR) following from a test patient group with successful recanalization (REC) and a test patient group with persistent occlusion (OCC). The difference and average are between the predicted volume and the follow-up volume. The continuous line represents the mean volume difference, which is positive for an overestimation of predicted volumes. The dashed lines represent the mean volume difference plus or minus 1.96 times the standard deviation.
